# A Novel Three-LncRNA Signature Predicting Tumor Recurrence in Nonfunctioning Pituitary Adenomas

**DOI:** 10.3389/fgene.2021.754503

**Published:** 2021-10-20

**Authors:** Sen Cheng, Jing Guo, Dawei Wang, Qiuyue Fang, Yulou Liu, Weiyan Xie, Yazhuo Zhang, Chuzhong Li

**Affiliations:** ^1^ Department of Neurosurgery, Beijing Tiantan Hospital Affiliated to Capital Medical University, Beijing, China; ^2^ Beijing Neurosurgical Institute, Capital Medical University, Beijing, China; ^3^ Beijing Institute for Brain Disorders Brain Tumor Center, Beijing, China; ^4^ China National Clinical Research Center for Neurological Diseases, Beijing, China

**Keywords:** non-functioning pituitary adenoma (NFPA), recurrence, long noncoding RNAs, signature, machine learning

## Abstract

The nonfunctioning pituitary adenoma (NFPA) recurrence rate is relatively high after surgical resection. Here, we constructed effective long noncoding RNA (lncRNA) signatures to predict NFPA prognosis. LncRNAs expression microarray sequencing profiles were obtained from 66 NFPAs. Sixty-six patients were randomly separated into a training (*n* = 33) and test group (*n* = 33). Univariable Cox regression and a machine learning algorithm was used to filter lncRNAs. Time-dependent receiver operating characteristic (ROC) analysis was performed to improve the prediction signature. Three lncRNAs (LOC101927765, RP11-23N2.4 and RP4-533D7.4) were included in a prognostic signature with high prediction accuracy for tumor recurrence, which had the largest area under ROC curve (AUC) value in the training/test group (AUC = 0.87/0.73). The predictive ability of the signature was validated by Kaplan-Meier survival analysis. A signature-based risk score model divied patients into two risk group, and the recurrence-free survival rates of the groups were significantly different (log-rank *p* < 0.001). In addition, the ROC analysis showed that the lncRNA signature predictive ability was significantly better than that of age in the training/testing/entire group (AUC = 0.87/0.726/0.798 *vs.* AUC = 0.683/0.676/0.679). We constructed and verified a three-lncRNA signature predictive of recurrence, suggesting potential therapeutic targets for NFPA.

## Introduction

Pituitary adenoma (PA) is a common and benign intracranial tumor that occurs in the pituitary gland (Fernandez et al., 2010; Ostrom et al., 2015). It can be divided into functioning and nonfunctioning pituitary adenoma (FPA and NFPA, respectively) according to the presence or absence of hormone oversecretion and/or related clinical symptoms, like hyperthyroidism, acromegalic features, and hyperprolactinemia (Moreno et al., 2005). NFPAs account for 14–54% of PAs, and the annual incidence is 0.65–2.34 cases/100,000 (Raappana et al., 2010; Tjörnstrand et al., 2014; Al-Dahmani et al., 2016; Day et al., 2016). Due to the lack of typical symptoms related to hormone hypersecretion, NFPA is usually detected based on symptoms caused by tumor pressure on surrounding structures, such as headaches or visual impairment, or found incidentally on imaging tests (Chen et al., 2011; Ntali and Wass, 2018). Surgical treatment is effective for NFPAs; however, total resection is not achievable for some tumors because they can invade the cavernous sinus or the area around the internal carotid artery (Meij et al., 2002; Shomali and Katznelson, 2002). Moreover, the recurrence rate of residual tumors reches 40 and 50% at 5 and 10 years, respectively, and even tumors that are completely resected have a recurrence rate of 10–20% after 5–10 years (Brochier et al., 2010; Chen et al., 2012; Sadik et al., 2017). Therefore, addressing the recurrence of NFPA is warranted. Currently, radiotherapy is considered to be effective in treating patients with residual or recurrent NFPA, although it may lead to progressive hypopituitarism and other long-term complications (Brada and Jankowska, 2008; Pollock et al., 2008). However, many questions remain about which subsets of NFPA patients are more likely to have recurrence and which subsets of residual tumors need to be further treated to prevent regrowth. Therefore, a method for predicting tumor recurrence after initial surgery is needed for early intervention.

Long noncoding RNAs (lncRNAs) are greater than 200 nt in length and have limited protein-coding ability (Moran et al., 2012). Emerging evidence suggests that lncRNAs regulate gene expression at the transcriptional and posttransciptional levels and that the dysfunction of lncRNAs contributes to the progression of many cancers, including PA (Poliseno et al., 2010; Wang and Chang, 2011; Huarte, 2015; Beylerli et al., 2020). Zhao et al. (2021) showed that downregulation of lncRNA PCAT6 could inhibit the proliferation, migration, viability, and invasion of PA cells by modulating the miR-139-3p/BRD4 axis . A study by D'Angelo et al. (2019) found that the lncRNA RPSAP52 promotes PA cell growth by acting as a microRNA (miRNA) sponge for HMGA proteins. The above studies verify that lncRNAs play a critical role in PA progression. Moreover, recent studies suggest that lncRNAs can be used to predict cancer prognosis and can as a signature in several cancers, such as oesophageal squamous cell carcinoma, gastric cancer, and hepatocellular carcinoma (Li et al., 2014; Zhu et al., 2016; Hong et al., 2020). However, the mechanism and prognostic value of lncRNAs in NFPA are still unclear. Therefore, it is necessary to find an appropriate lncRNA signature to accurately predict the recurrence of NFPA patients after surgery to provide early intervention.

In this study, tumor recurrence refers to regrowth of residual tumor cells and tumor relapse after total resection. We analyzed the expression of lncRNAs in 66 NFPA patients through microarray sequencing and identified genes associated with tumor recurrence. We aimed to develop and validate a useful multi-lncRNA prediction model that may be used to evaluate recurrence and guide treatment after surgical resection in patients with NFPA.

## Methods

### Patients and Samples

From October 2007 to July 2014, patients who were diagnosed with NFPA and underwent surgical resection at Beijing Tiantan Hospital were included in this study (*n* = 66). The mean age of these 66 patients was 51.5 years (range, 25–73), there were 34 males and 32 females, and the median follow-up was 76.5 months (range, 5–122). The clinical and pathological characteristics of all the patients are shown in Supplementary Table S1. Cavernous sinus (CS) invasion was defined by the Knosp grading scale (grade 3 or 4) on preoperative enhanced magnetic resonance imaging (MRI) (Knosp et al., 1993). Postoperative tumor recurrence was defined as recurrence identified from any direction on enhanced MRI from the day of surgery to the end of the follow up; the maximum tumor diameter needed to increase by > 2 mm. According to tumor size, NFPA were divided into microadenoma (<10 mm in diameter), macroadenoma (≥10 mm) and giant adenoma (≥40 mm). The local Ethics Committee approved this study, and informed consent was obtained from each subject.

### Total RNA Extraction

According to the instructions provided, total RNA was extracted and purified from collected samples using the phenol-free mirVana™ miRNA Isolation Kit (Cat # AM1561; Ambion; Thermo Fisher Scientific, Inc.). A Thermo Scientific^TM^ NanoDrop 2000 was used to quantify and assess purity of the extracted RNA.

### RNA Microarray Analysis

RNA samples were used to generate fluorescence-labeled cRNA targets for the SBC human ceRNA array V1.0 (4 × 180 K) and were subsequently hybridized with slides and scanned in an Agilent Microarray Scanner (Agilent Technologies, Santa Clara, CA, United States) to obtain the data. The raw data was extracted using feature extraction software 10.7 (Agilent Technologies, Inc.). Then, the quantile algorithm provided by the “limma” package (http://bioconductor.org/packages/limma/) of the R program was used to normalize the data.

### Identification of Prognostic LncRNAs

The “sample” function of R progrom (www.r-project.org/) was used to randomly divided 66 NPFA patients into a training set (*n* = 33) and a testing set (*n* = 33). In the training group, univariable Cox proportional hazards regression analysis was performed to determine the association between recurrence-free survival (RFS) and lncRNA expression in each patient. We used a machine learning approach, random survival forests-variable hunting (RSFVH) algorithm, to narrow the scope of the gene set through an iteration procedure, discarding the bottom quarter of lncRNAs (the least important lncRNAs) at each step. In total, nine lncRNAs were selected (Mogensen et al., 2012; Li et al., 2014; Ishwaran and Lu, 2019).

### Construction of Prognostic LncRNA Signature

The selected lncRNAs was used to construct a risk prediction score model as follows (Ritchie et al., 2015; Guo et al., 2016).

#### Risk Score (RS) = ∑ ^N^i = 1 (Explg *Coef)

In this formula, N represent the number of prognostic lncRNA, Explg represents the expression value of lncRNA, and Coef represents the estimated regression coefficient of the lncRNA in the univariable Cox regression analysis.

Since the nine selected lncRNAs could form 2^9^–1 = 511 combinations or signature, each patient received 511 risk scores. Then, in the training dataset, the sensitivity and specificity of the 511 signatures were analyzed by the time-dependent receiver operating characteristic (ROC) curves. The prognostic signature was obtained by comparing the area under the ROC curve (AUC) values.

### Validation the Reliability of Microarray Data by RT-PCR

To verify the existence of the lncRNA signature, twelve samples were randomly selected from the entire group for RT-PCR and agarose gel electrophoresis. LncRNA reverse transcription was performed using a High Capacity cDNA Reverse Transcription Kit (0049472, Thermo Fisher). Next, PCR was performed using I-5TM High-Fidelity Master Mix (I5HM, 200MCLAB). PCR was conducted as follows: 2 min of initial denaturation at 98°C, 32 cycles of 10 s at 98°C, 58°C for 10 s and 72˚C for 10s, and final extension step for 5 min at 72˚C. GAPDH was used as an internal control gene. The PCR products were run on 2% agarose gel and visualized using a UV transilluminator. The primer sequences are presented in Supplementary Table S2.

### Statistical Analysis

The survival distribution of different groups was evaluated and compared using Kaplan-Meier survival analyses and two-sided log-rank tests. The chi-square test was used to analyzed the associations with clinical signatures. *p* < 0.05 was considered to indicate statistical significance. All analyses were performed using R program 3.6.1. The packages were downloaded from Bioconductor, including the survival, ROC, and randomForestSRC packages.

### Functional Enrichment Analysis of LncRNAs With Prognostic Value

To investigate the potential function of the lncRNAs in the signature, Pearson correlation tests were used to identified protein-coding genes (PCGs) coexpressed with the prognostic lncRNAs. The genes with a *p* < 0.05 and an absolute value of the Pearson coefficient > 0.6 were selected for Gene Ontology (GO) (Ashburner et al., 2000; The Gene Ontology, 2017) and Kyoto Encyclopedia of Genes and Genomes (KEGG) (Kanehisa and Goto, 2000; Kanehisa et al., 2016; Kanehisa et al., 2017) enrichment analyses. The GO and KEGG analyses were performed with the clusterProfiler package (Yu et al., 2012) of the R program.

## Results

### Identification of LncRNA Signatures for the Prediction of NFPA Recurrence

**FIGURE 1:**
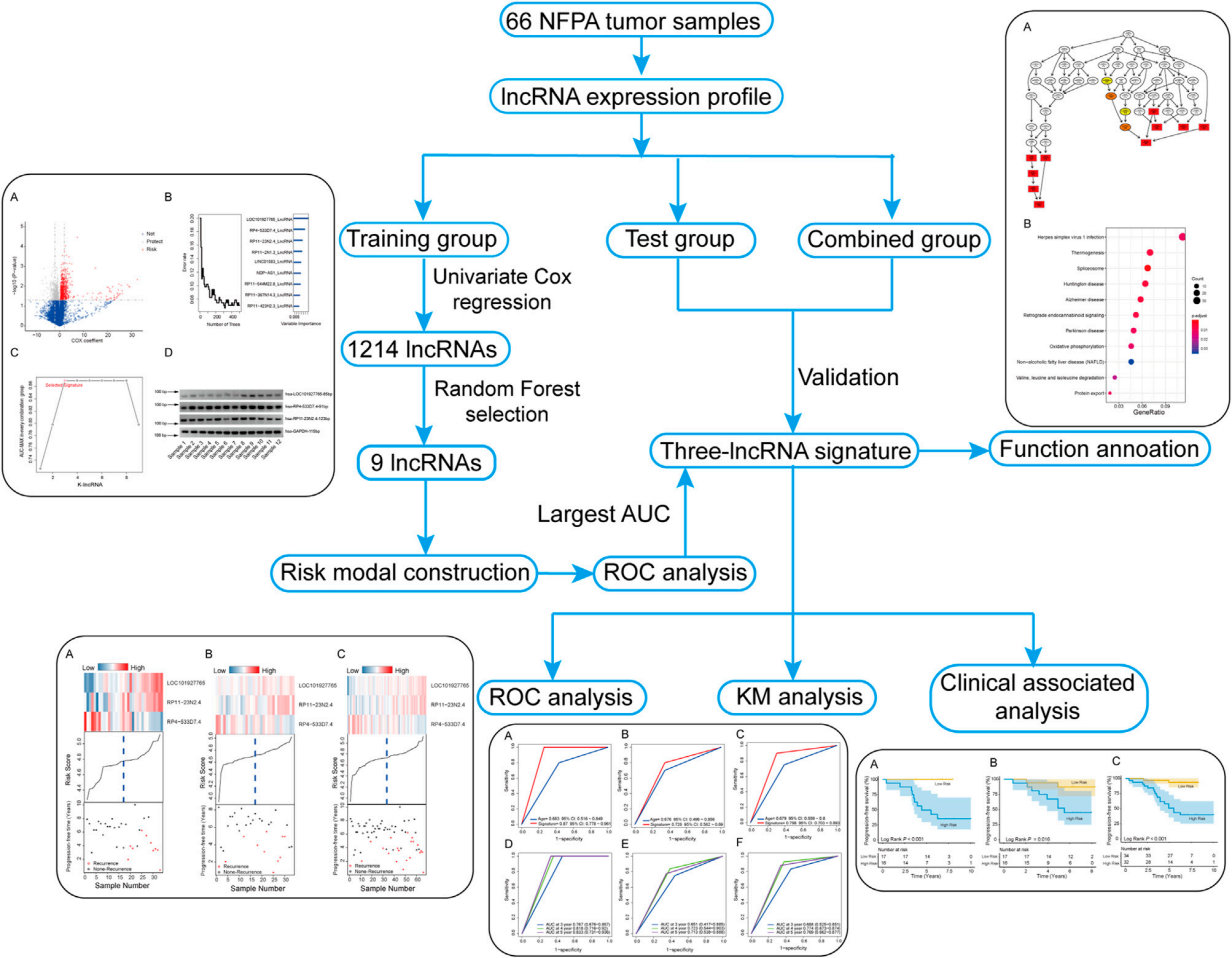
Flowchart of the study.

A total of 19,741 lncRNAs were extracted from the 66 NFPA expression profiles. The flow chart of this study is shown in Figure 1. The patient information of all patients is summarized in Table 1.

**TABLE 1:** Clinical Data of the included tumors.

	Entire set (n)	Training set (n)	Test set (n)
Gender			
Male	32	18	14
Female	34	15	19
Age (years)			
≤52	38	19	19
>52	28	14	14
Tumor size classification			
Macro	47	24	23
Giant	19	9	10
CS Invasion			
Yes	38	20	18
No	28	13	15
Headache			
Yes	31	14	17
No	35	19	16
Vision and visual field disorders			
Yes	50	26	24
No	16	7	9
Recurrence			
Yes	20	10	10
No	46	23	23

CS, cavernous sinus; Giant, giant adenoma; Macro, macroadenoma.

**FIGURE 2:**
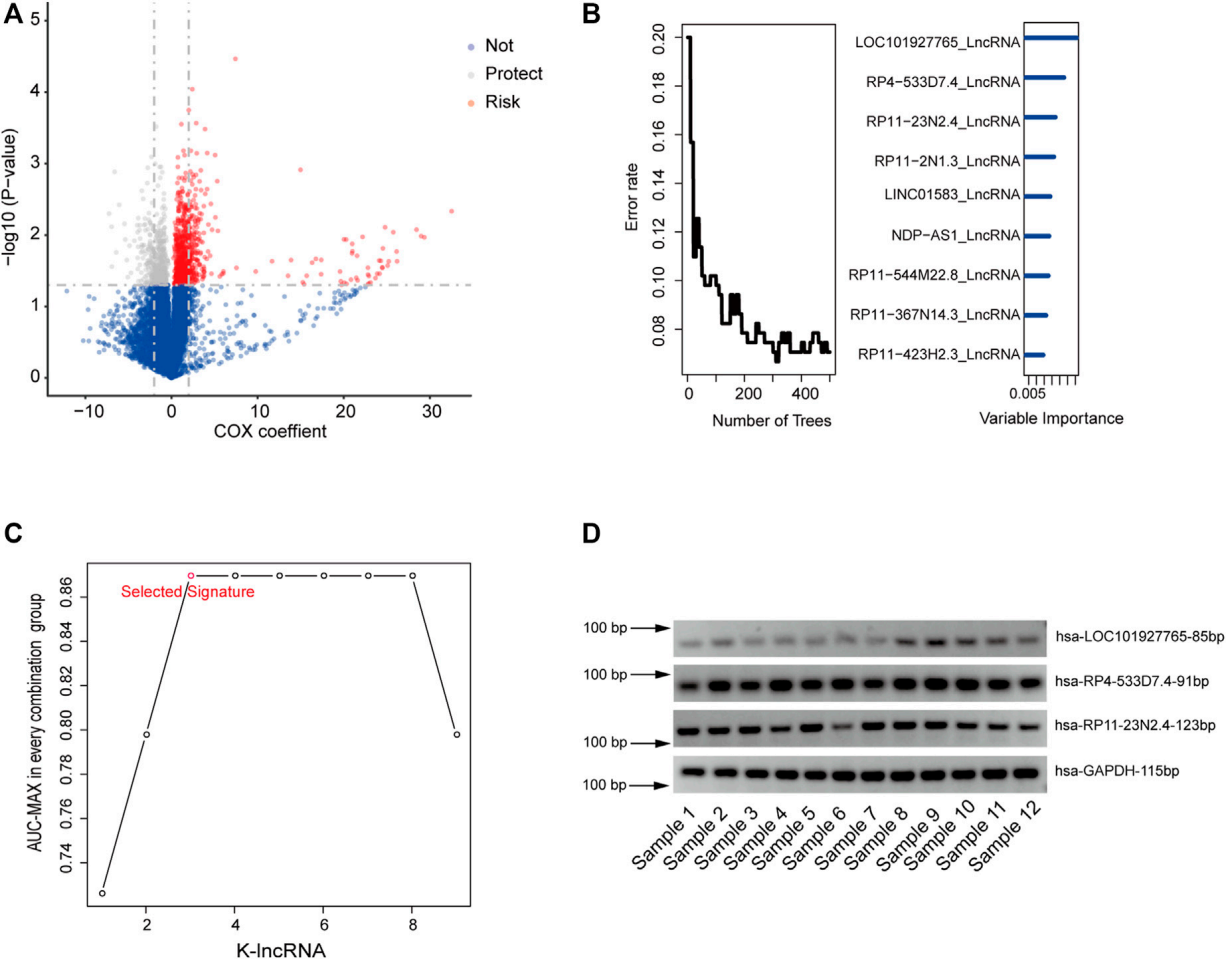
Identifying the LncRNA signature in the training dataset. **(A)**, Univariate Cox proportional hazards regression analysis of the lncRNA expression profiling data in the training dataset. **(B)**, Identifying the lncRNAs by RFSC algorithm. **(C)**. The AUC of all 511 signatures were calculated and the nine highest AUC for k = 1, 2…9 is shown in the plot by ROC analysis for the lncRNA signatures predicting model in the training dataset. **(D)**, Agarose electrophoresis of selected lncRNAs PCR products.

Initially, in the training set, univariate Cox proportional hazards regression analysis was used to obtain RFS-related lncRNAs. The 1,214-lncRNA set was identified using recurrence as the dependent variable, and the signature was significantly associated with patient recurrence (Supplementary Table S3, *p* value < 0.05, Figure 2A).

Secondly, to further reduce the number of prognostic lncRNAs, the random forest supervised cassification (RFSC) algorithm was employed to analyze the 1,214-lncRNA set, and the nine lncRNAs most related to recurrence were obtained according to the permutation important score calculated with the RFSC algorithm (Figure 2B; Supplementary Figure S1).

Thirdly, based on the nine types of lncRNA, we constructed a risk-score model of 2^9^–1 (511) types of lncRNA set combinations, which contained different lncRNA numbers from 1 to 9. To screen for a better prediction signature, we conducted a time-dependent ROC analysis that used recurrence status as a lable and signature risk scores as a variable in the training group and compared the sensitivities and specificities (Supplementary Table S4).

According to the AUC values of all 511 signatures (Supplementary Table S4), we identified the lncRNA combination composed of LOC101927765, RP11-23N2.4, and RP4-533D7.4 as the most promising, as it had strong ability to predict recurrence and the smallest node and the largest AUC value of 0.87 (Figure 2C; Table 2). RT-PCR was used to confirm the reliability of microarray sequencing. Consisting with the microarray data, the three lncRNAs were detected in 12 tumor tissues (Figure 2D), which revealed that the lncRNA are stable and can be used as prognostic maker.

**TABLE 2:** Identities of PCG and lncRNAs in the prognostic expression signature and their univariable cox association with prognosis.

Gene symbol	Coefficient^a^	*p* Value^a^	Gene expression level association with poor prognosis
LOC101927765	3.406	0.001	high
RP11-23N2.4	1.895	0.007	high
RP4-533D7.4	−3.440	0.002	low

a Derived from the univariable Cox regression analysis in the training set.

The risk score of the signature was calculated as follows: risk score = (3.41 × expression value of LOC101927765) + (1.90 ×  expression value of RP11-23N2.4) + (−3.43 ×  expression value of RP4-533D7.4).

### Validation the Prediction Ability of the Three LncRNA Signature

**FIGURE 3:**
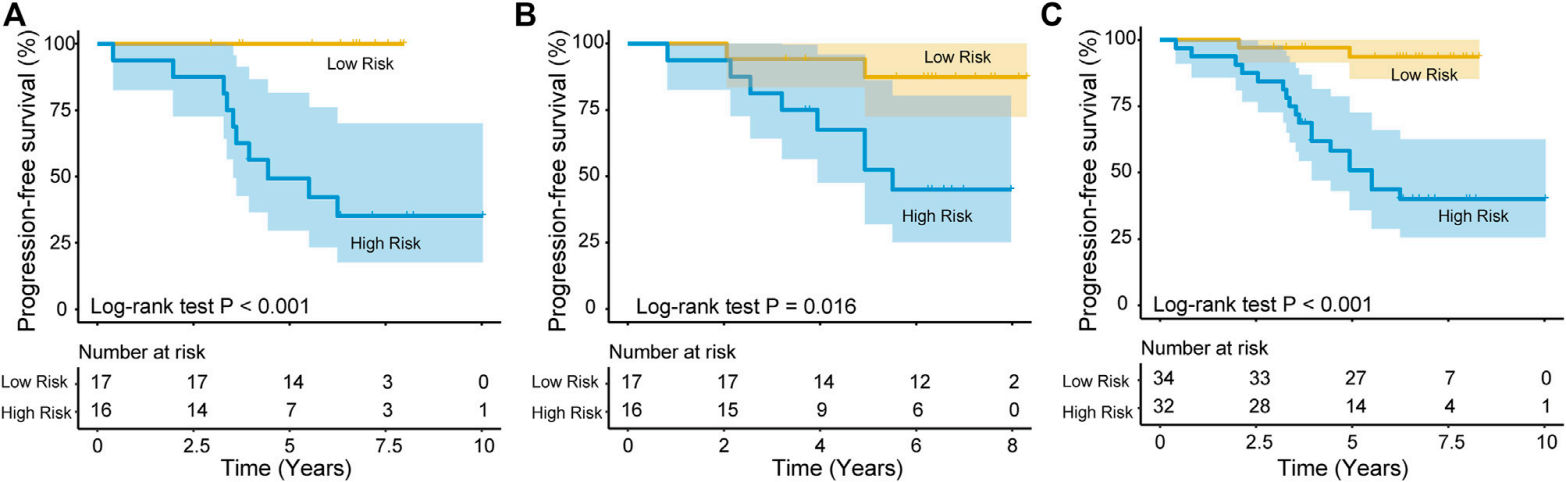
The lncRNA signature for predicting recurrence PFS of patients with NFPA. Kaplan–Meier survival curves of patients classified into high- and low-risk groups using the lncRNA signature in the training **(A)**, test **(B)** and entire dataset **(C)**.

Each patient obtains a risk score according to the risk score model. Then, the patients from the training group were divided into a high-risk group (*n* = 16) and a low-risk group (*n* = 17) based on the cutoff point, which was the median risk score. Kaplan-Meier survival analysis was performed to determined the difference in RFS between the two risk groups. The median RFS time was significantly shorter in the high-risk group (4.44 years) than in the low-risk group (6.74 years) (*p* < 0.001; log-rank test, Figure 3A). Moreover, the recurrence rate of the high-risk group was higher than that of the low-risk group (>60% *vs*. < 1%). In a similar manner, patients from the test group were also divided into two risk groups. The results of Kaplan-Meier analyses for the high-risk (*n* = 16) and low-risk (*n* = 17) groups in the test dataset were plotted and are shown in Figure 3B (median RFS time: 5.51 *vs*. 6.82 years; log-rank test, *p* = 0.016), and the RFS rates were approximately 52.25 and 87.40%, respectively. In addition, patients in the entire group were similary divided into high-risk (*n* = 32) and low-risk (*n* = 34) groups, and Kaplan-Meier analysis further confirmed the ability of the lncRNA signature to predict recurrence (median PFS time: 4.97 *vs*. 6.79 years; log-rank test, *p* < 0.001, Figure 3C).

**FIGURE 4:**
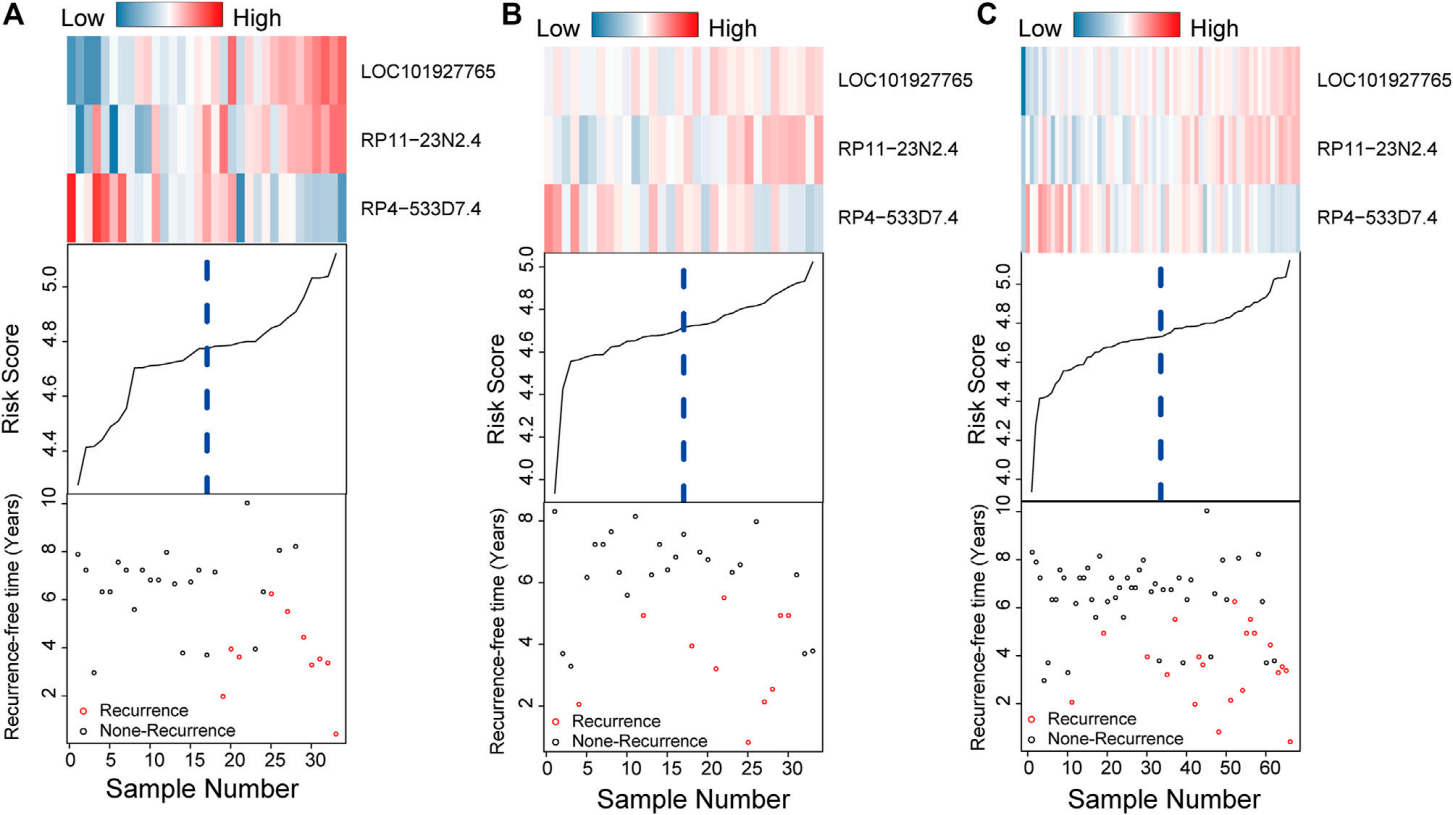
Risk score distribution, survival status and gene expression patterns for patients in high- and low-risk cluster grouped by the three-lncRNA signature in the training **(A)**, testing **(B)**, and independent datasets **(C)**.

Figures 4A–C intuitively shows the risk score, survival status and expression pattern of lncRNAs in the training, testing, and independent datasets. For patients with low risk scores in the three datasets, RP4−533D7.4 was highly expressed, while LOC101927765 and RP11-23N2.4 was expressed at low levels; the opposite patterns for each lncRNA were seen in patients with high risk scores.

### The Value of the LncRNA Signature is Independent of Traditional Clinical Features

After proving the recurrence prediction ability of the lncRNA signature, we explored the correlation between the signature and clinical characteristics in the entire dataset (*n* = 66) to understand the clinical significance of the lncRNA signature.

Table 3 shows that there was an association between the lncRNA signature and age in the entire group (chi-suqre test, *p* = 0.03, Table 3). In addition, we further assessed whether the prognostic value of the three-lncRNA signature was independent of other clinical factors. Univariate and multivariate Cox regression analyses of factors including age, sex, tumor size classification, CS invasion, and the signature were performed. In the entire dataset, age (HR = 0.33, 95% CI = 0.12–0.93, *p* = 0.04) and the signature risk score (HR = 1.50, 95% CI = 1.24–1.82, *p* < 0.001) were significantly associated with the RFS of patients (Table 4). Moreover, the three-signature score was also an indenpent prognostic factor associated with RFS in the training (HR = 2.06, 95% CI = 1.36–3.12, *p* < 0.001) and test set (HR = 6.96, 95% CI = 1.21–40.16, *p* = 0.03). Hence, the results indicate that the three-lncRNA signature is an independent prognostic factor for NFPA RFS.

**TABLE 3:** Association of the signature with clinicopathological characteristics in Pituitary adenoma patients.

Variables	Training			Test			Entire		
	Low risk	High risk	P	Low risk	High risk	P	Low risk	High risk	P
Sex			1.00			0.36			0.62
Female	8	7		8	11		16	18	
Male	9	9		9	5		18	14	
Age			0.21			0.12			0.03
≤52	7	11		5	10		12	21	
>52	10	5		12	6		22	11	
Tumor size classification			0.50			0.40			0.70
Giant	6	3		5	5		11	8	
Macro	11	13		12	11		23	24	
Invasion			1.00			0.21			0.43
No	6	6		10	5		16	11	
Yes	11	10		7	11		18	21	

Data were analyzed using the Chi-squared test; *p*-value < 0.05 was considered to indicate a statistically significant difference.

**TABLE 4:** Univariable and multivariable Cox regression analysis of the signature and survival of NFPA patients in the training, test group and entire group.

Variables		Univariable analysis				Multivariable analysis			
		HR	95% CI of HR		P	HR	95% CI of HR		P
			Lower	Upper			Lower	Upper	
Training set (*n* = 33)									
Age	>52 *vs*.≤52	0.23	0.05	1.09	0.06	0.18	0.03	1.08	0.06
Sex	Male *vs*. Female	1.05	0.29	3.74	0.94	0.73	0.17	3.11	0.68
Tumor size classification	Macro *vs*. Giant	1.10	0.23	5.18	0.91	1.19	0.18	7.72	0.85
CS invasion	Yes *vs*. No	1.40	0.36	5.40	0.63	1.37	0.28	6.59	0.70
Signature	High risk *vs*. low risk	2.03	1.40	2.94	<0.001	2.06	1.36	3.12	<0.001
Test set (*n* = 33)									
Age	>52 *vs*.≤52	0.35	0.09	1.36	0.13	0.62	0.15	2.67	0.52
Sex	Male *vs*. Female	0.53	0.14	2.03	0.35	0.78	0.17	3.72	0.76
Tumor size classification	Macro *vs*. Giant	0.31	0.09	1.08	0.07	0.15	0.02	0.88	0.04
CS invasion	Yes *vs*. No	2.05	0.53	7.92	0.30	0.43	0.06	3.29	0.42
Signature	High risk *vs*. low risk	5.49	1.16	14.92	0.03	6.96	1.21	40.16	0.03
Entire set (*n* = 66)									
Age	>52 *vs*.≤52	0.29	0.10	0.79	0.02	0.33	0.12	0.93	0.04
Sex	Male *vs*. Female	0.75	0.31	1.81	0.52	0.89	0.36	2.18	0.80
Tumor size classification	Macro *vs*. Giant	1.21	0.82	1.78	0.33	0.59	0.21	1.67	0.32
CS invasion	Yes *vs*. No	1.72	0.66	4.48	0.26	1.23	0.42	3.54	0.71
Signature	High risk *vs*. low risk	1.49	1.24	1.80	<0.001	1.50	1.24	1.82	<0.001

CS, cavernous sinus; Giant, giant adenoma; Macro, macroadenoma.

### Comparison of the Predictive Power of the LncRNA Signature and Age

**FIGURE 5:**
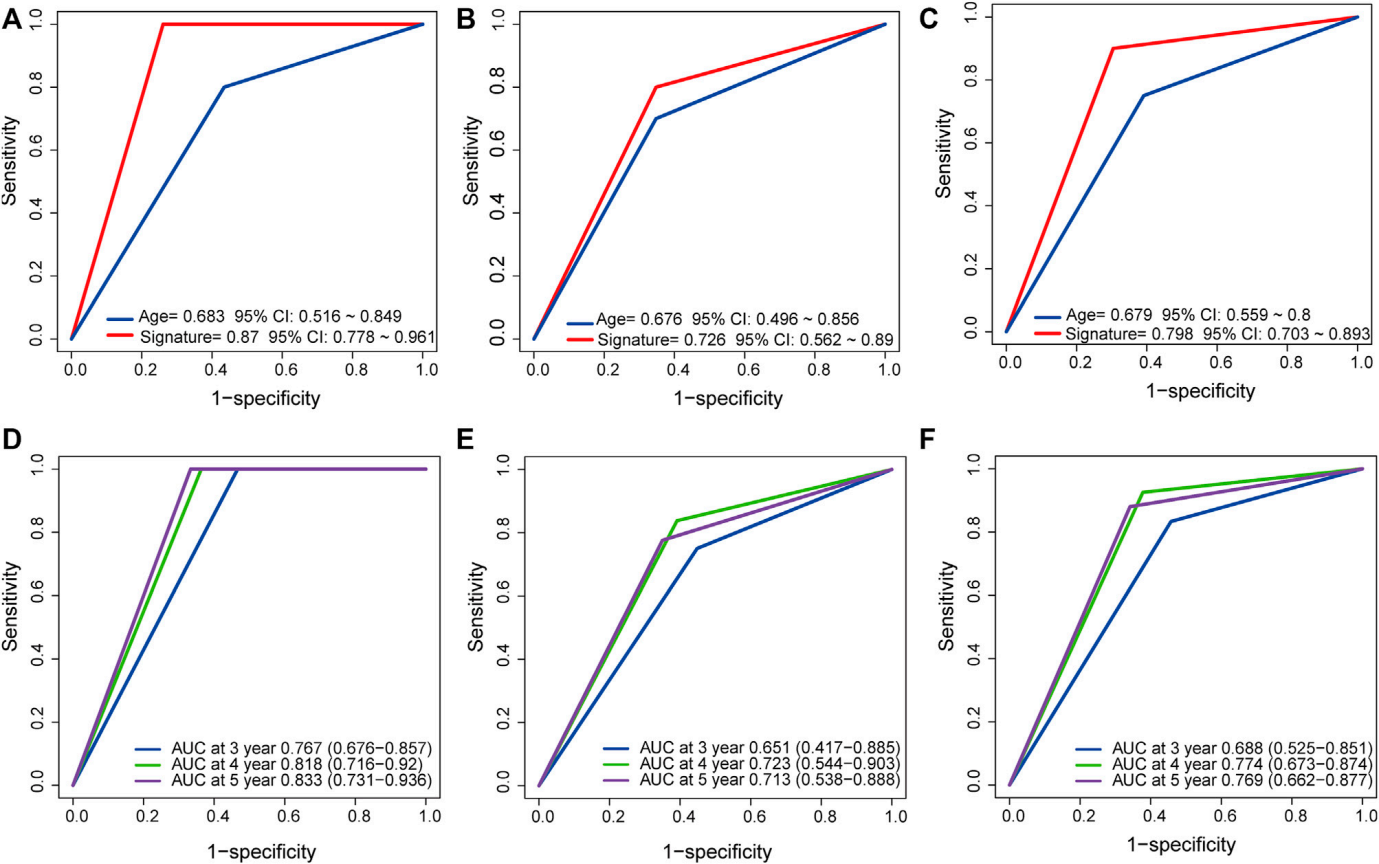
Compare prediction power of the lncRNA signature to that of Age by ROC in the training, test, and entire dataset **(A, B, C)** and TimeROC analysis for the signature at 3,4, and 5 years in the three sets **(D, E, F)**.

It has been reported that age is associated with a risk of tumor recurrence (Losa et al., 2008). ROC analysis was performed to determine the predictive power of the lncRNA signature and age. The results showed that in the training/testing/entire group, the AUC values of the lncRNA signature were lager those of age (AUC = 0.87/0.726/0.798 *vs.* AUC = 0.683/0.676/0.679, Figures 5A–B), indicating that the signature had high accuracy and important clinical significance. In addition, time-dependent ROC analysis was performed on the three datasets to further understand the signature prediction capabilities for 3-, 4- and 5 year RFS. The signature AUC values in the training/test/entire group at 3, 4, and 5 years, as shown in Figures 5D–F, indicated a strong predictive power of the signature for RFS (AUC = 0.767/0.818/0.833, 0.651/0.723/0.713, and 0.688/0.774/0.769, respectively).

### Functional Enrichment Analysis of Genes Associated with the Prognostic LncRNAs in the Signature

**FIGURE 6:**
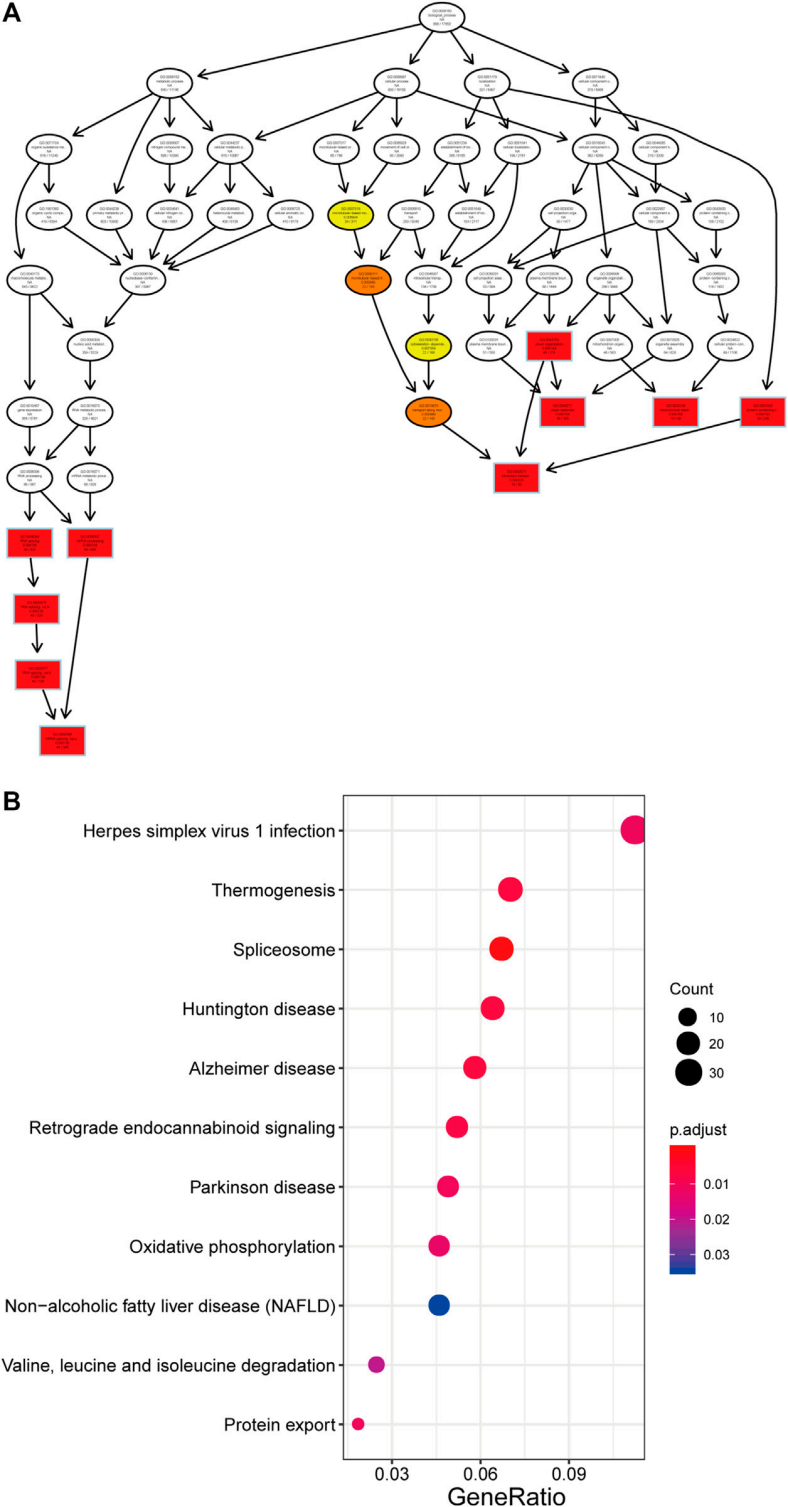
Function of the three lncRNA for GO **(A)** and KEGG **(B)** analysis by clusterProfiler.

The PCGs correlated with the lncRNAs in our prognostic signature were obtained by Pearson correlation analysis in all 66 patients, and their potential biological function were explored. The expression of 1,056 PCGs was highly correlated with that of at least one of the LncRNAs (Pearson correlation coefficient > 0.60, *p* < 0.05, Supplementary Table S5). Next, we performed GO and KEGG analyses and found these the genes were enriched in 99 different terms (Supplementary Table S6), such as mRNA processing, RNA splicing and oxidative phosphorylation (Figure 6).

## Discussion

The prevalence of NFPA ranges from 7 to 41.3 cases per 100,000 population, and it is the second most common type of adenomas after prolactinomas (Ntali and Wass, 2018). Despite NFPA being a histologically benign tumor and advances in endosopic techniques, the recueence rate of NFPA is relatively high (Batista et al., 2018). Therefore, it is necessary to accurately predict tumor recurrence after NFPA surgery to obtain the most effective and accurate treatment plan. Herein, we constructed a three-lncRNA signature to predict the prognostic of NFPAs and verifies its predictive power.

First, we obtained 19,741 lncRNA expression profiles by sequencing 66 NFPAs and identified 1,214 lncRNAs that were significantly related to RFS in NFPA in the training set. RSFVH algorithm, a machine learning method, was used to narrow down the number of RFS-related lncRNAs to 9. A three-lncRNAs (LOC101927765, RP11-23N2.4, and RP4-533D7.4) signature with the highest AUC value of 511signatures, which contained combinations of 1–9 different lncRNAs, was identified. The risk model of the signature was constructed basde on the three lncRNAs. Second, patients were divided into two risk group in the training and testing sets, and the recurrence prediction power was validated by Kaplan-Meier analysis. Third, the three-lncRNA signature-based risk score was identified as a prognostic factor independent of clinical features like sex, tumor size classification, CS invasion. Age is a controversial factor related to recurrence in NFPA. Batista et al. (2018) showed that recurrence of NFPA was not associated with age while Subramanian and indicated that older age at surgery was related to a lower risk of recurrence (Lyu et al., 2021; Subramanian et al., 2021). Even so, the ROC analysis showed that the predictive ability of the three-lncRNA signature was better than that of age. Finally, we explore the potential biological function of the three lncRNAs through functional enrichment analysis of coexpressed PCGs, which were identified as related to the three lncRNAs by Perason correlation analysis.

In recent years, lncRNAs has been considered potential prognostic markers and therapectic targets for cancers (Sanchez Calle et al., 2018; Zhang et al., 2021). Liu et al. (2020) found that lncCSMD1-1 is overexpressed in hepatocellular carcinoma (HCC) and interacts with the MYC protein to promote tumor progression, suggesting that it may serve as a prognostic marker for HCC. The lncRNA PiHL (RP11-382A18.2) is upregulated in colorectal cancer (CRC), and its upregulation is an independent predictor of poor CRC prognosis (Deng et al., 2020). In addition, lncRNA also play a crucial role in PA progression. Wang et al. (2019) demonstrated that the lncRNA clarin 1 antisense RNA 1 (CLRNA-AS1) was expressed at low levels in prolactinoma and inhibited cell proliferation and autophagy Moreover, lncRNA-H19 is downregulated in PA and negatively correlated with tumor progression (Wu et al., 2018). Therefore, lncRNAs may be developed into a prognostic makers of PA. Recently, an increasing number of studies have identified several lncRNAs that can be studied to predict cancer prognostic. Meng et al. (2014) identified four lncRNA genes (U79277, AK024118, BC040204, AK000974) that can be used to predict breast cancer survival. Jiang et al. (2020) found that three-lncRNA (LINC02434, AL139327.2, and AC126175.1) could be used to predict prognosis in head and neck squamous cell cancer However, these studies did not confirm the reliability of the lncRNAs in tumor samples. In the present study, to avoid false positives in sequencing data, RT-PCR was performed to verify the reliability of the three lncRNA.

There are some limitations in this study that need to be acknowledged. First, potential lncRNAs may have been overlooked because the study only included 19,741 lncRNAs, which is only a small fraction of human lncRNAs. Second, the construction and evaluation of the model were based on the limited NFPAs samples, and more external samples are needed to verify the prediction power. Third, further *in vivo* and *in vitro* experiment need to be performed to elucidate the mechanisms and potential functions of the three lncRNAs.

In summary, we constructed a three-lncRNAs signature that could serve as a precise predictive biomarker for NFPAs. In addition, patients identified by the 3-lncRNA signature to be at high risk of NFPA after surgery could benefit from early and accurate intervention.

## Data Availability

The original contributions presented in the study are included in the article/Supplementary Material, further inquiries can be directed to the corresponding author.
