# Supplementary figures and images for: A Novel Three-LncRNA Signature Predicting Tumor Recurrence in Nonfunctioning Pituitary Adenomas
Source: Front Genet. 2021 Oct 20;12:754503. doi: 10.3389/fgene.2021.754503 (PMC8564111; doi:10.3389/fgene.2021.754503)

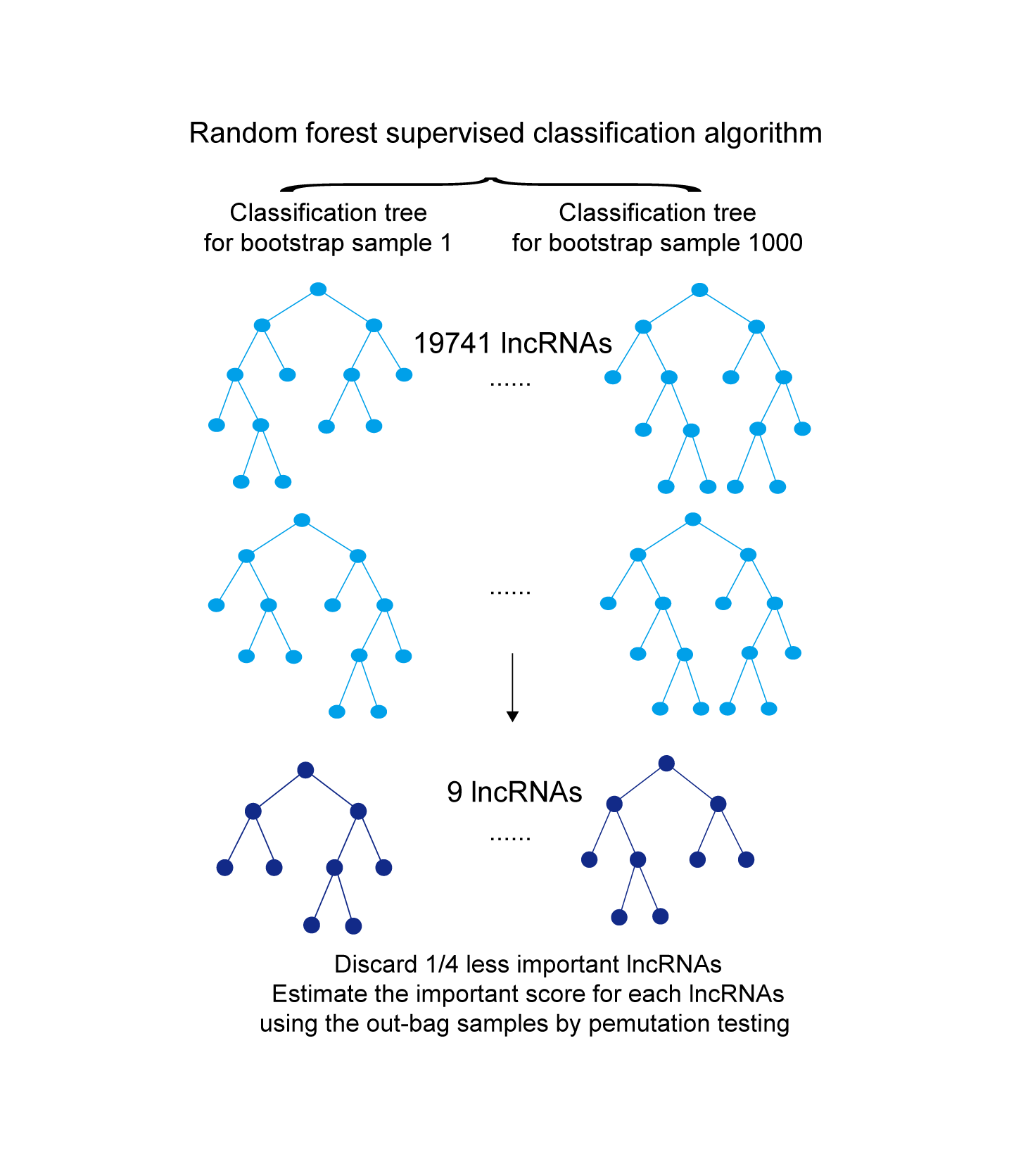

Supplement: Supplementary file 5 [file Image1.TIF]

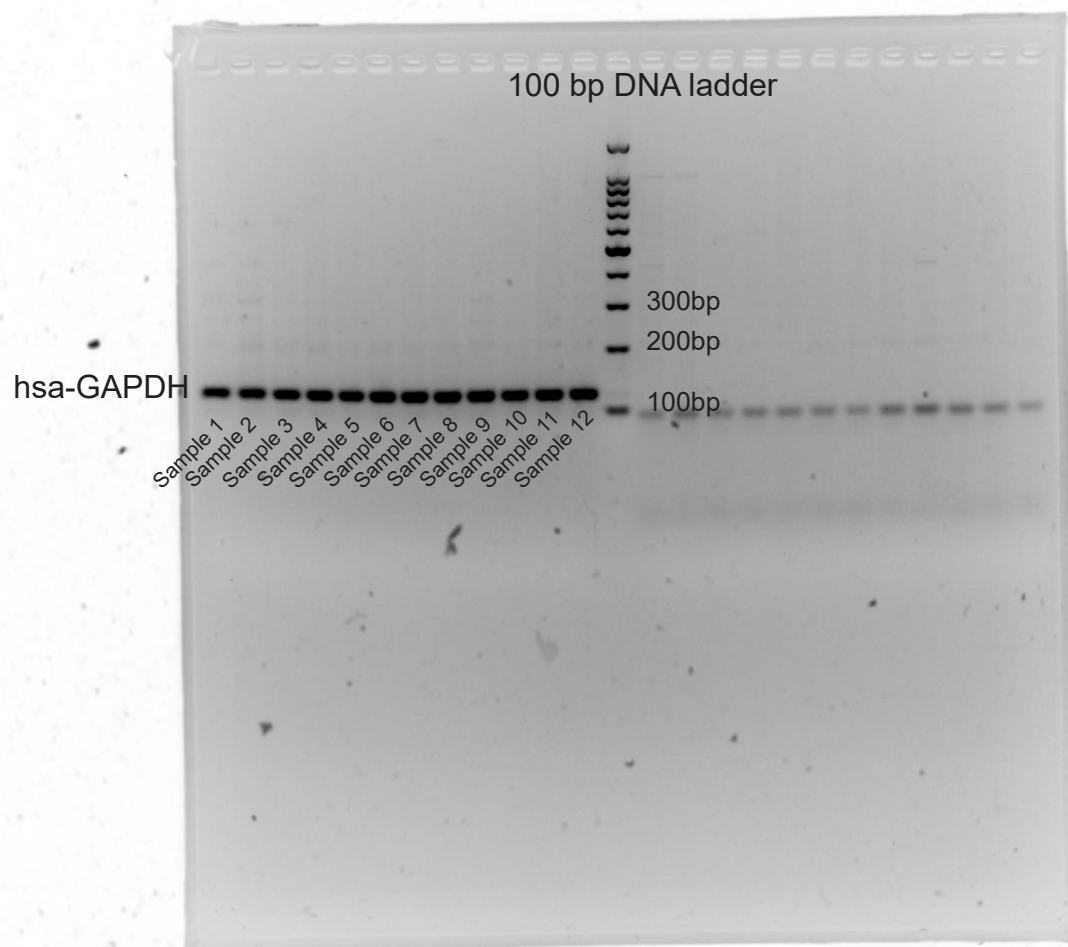

hsa-LOC101927765

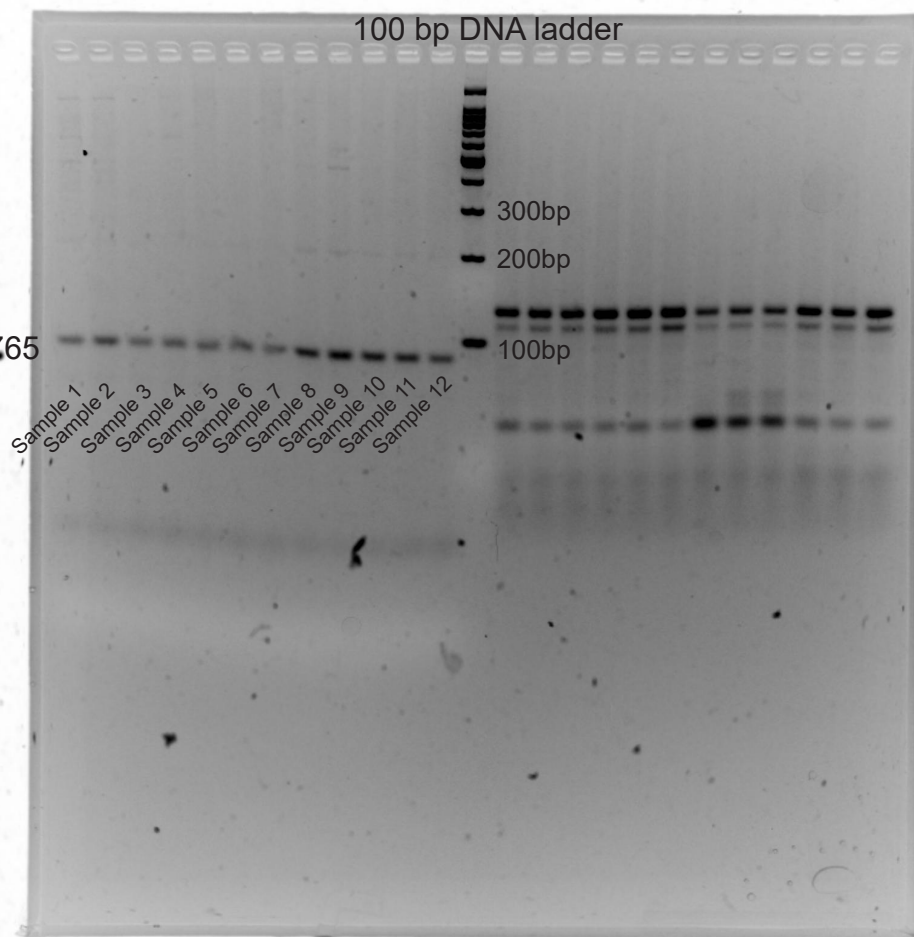

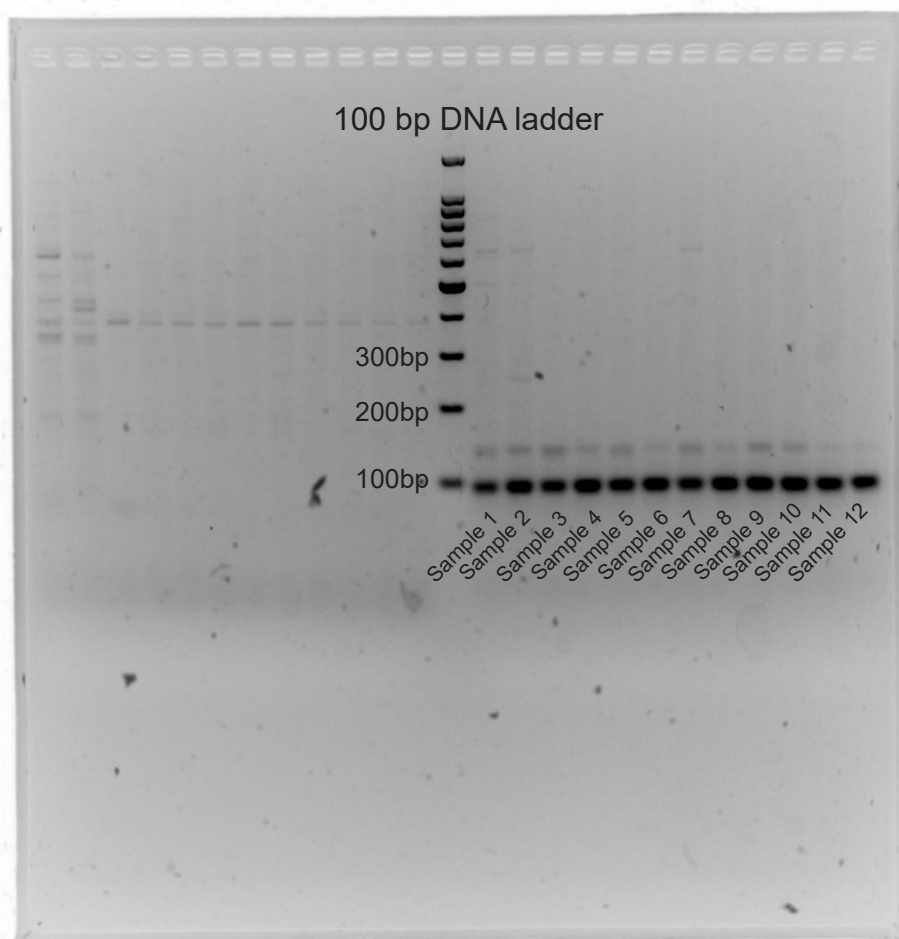

hsa-RP4-533D7.4

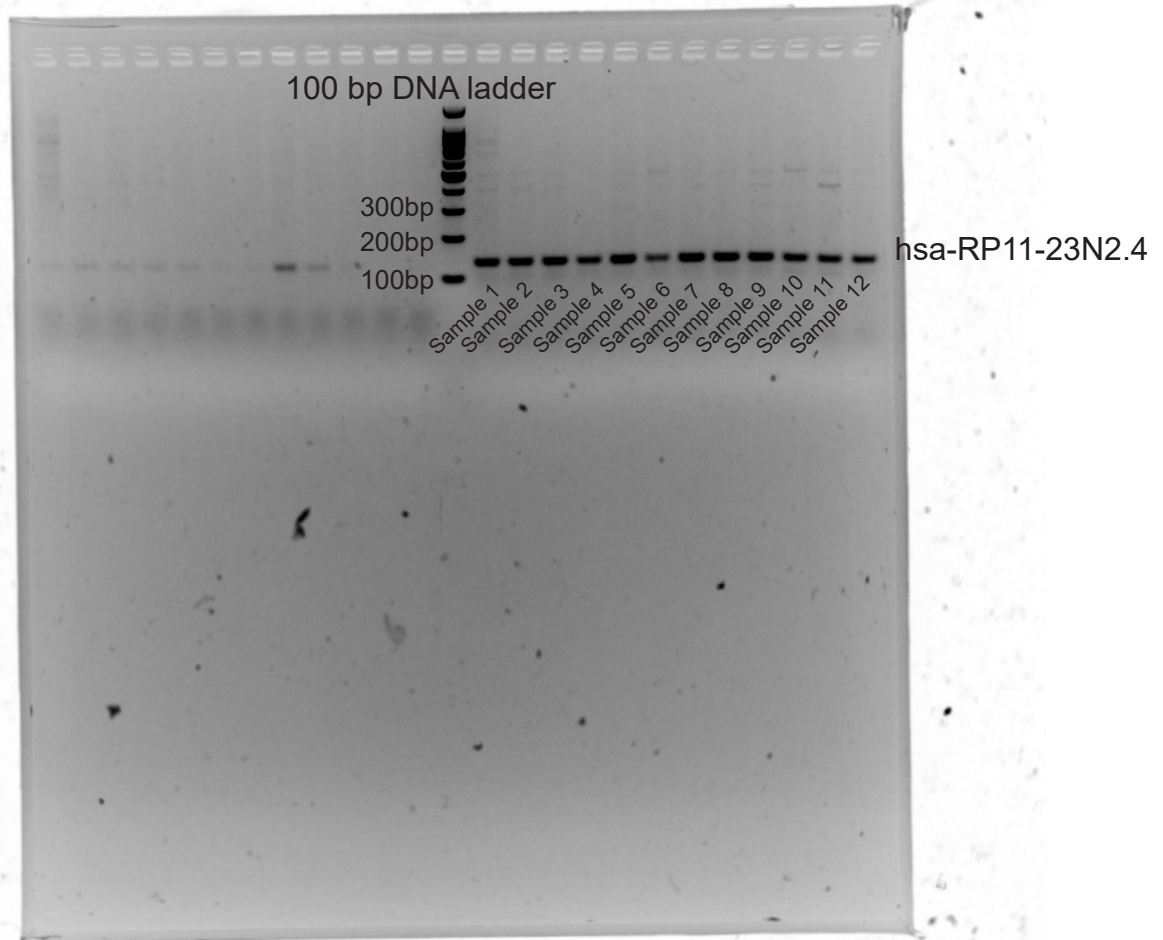

Supplement: Supplementary file 8 [file DataSheet1.PDF]
