# Supplementary material for: A Novel Three-LncRNA Signature Predicting Tumor Recurrence in Nonfunctioning Pituitary Adenomas
Source: Front Genet. 2021 Oct 20;12:754503. doi: 10.3389/fgene.2021.754503 (PMC8564111; doi:10.3389/fgene.2021.754503)
Supplement: Supplementary file 7 [file Table2.DOCX]

**Table S2: Primers used for RT-PCR**

| Gene | Forward primer (5′-3′) | Reverse primer (3′-5′) |
| --- | --- | --- |
| LOC101927765 | GGCAGGGGAGTTGGGAAAATAA | GAAGCAACTCTCTTCATCCCTTT |
| RP4-533D7.4 | ACATACTCGCACTCACAGTGG | GTGTCCTCAGGCTTAGTGGAG |
| RP11-23N2.4 | ATCAATGGCCACAGCACTCA | TCCCCTCTCCCTCAAGATACA |
| GAPDH | GCCATCACTGCCACTCAGAAGA | ATGACCTTGCCCACAGCCTTG |
